# The Association between Asthma and Allergic Symptoms in Children and Phthalates in House Dust: A Nested Case–Control Study

**DOI:** 10.1289/ehp.7187

**Published:** 2004-07-15

**Authors:** Carl-Gustaf Bornehag, Jan Sundell, Charles J. Weschler, Torben Sigsgaard, Björn Lundgren, Mikael Hasselgren, Linda Hägerhed-Engman

**Affiliations:** ^1^Swedish National Testing and Research Institute, Borås, Sweden; ^2^Technical University of Denmark, Lyngby, Denmark; ^3^Public Health Science, Karlstad University, Karlstad, Sweden; ^4^University of Medicine and Dentistry New Jersey–Robert Wood Johnson Medical School and Rutgers University, Piscataway, New Jersey, USA; ^5^Aarhus University, Aarhus, Denmark

**Keywords:** allergy, asthma, BBzP, children, DEHP, homes, phthalates

## Abstract

Global phthalate ester production has increased from very low levels at the end of World War II to approximately 3.5 million metric tons/year. The aim of the present study was to investigate potential associations between persistent allergic symptoms in children, which have increased markedly in developed countries over the past three decades, and the concentration of phthalates in dust collected from their homes. This investigation is a case–control study nested within a cohort of 10,852 children. From the cohort, we selected 198 cases with persistent allergic symptoms and 202 controls without allergic symptoms. A clinical and a technical team investigated each child and her or his environment. We found higher median concentrations of butyl benzyl phthalate (BBzP) in dust among cases than among controls (0.15 vs. 0.12 mg/g dust). Analyzing the case group by symptoms showed that BBzP was associated with rhinitis (*p* = 0.001) and eczema (*p* = 0.001), whereas di(2-ethylhexyl) phthalate (DEHP) was associated with asthma (*p* = 0.022). Furthermore, dose–response relationships for these associations are supported by trend analyses. This study shows that phthalates, within the range of what is normally found in indoor environments, are associated with allergic symptoms in children. We believe that the different associations of symptoms for the three major phthalates—BBzP, DEHP, and di-*n*-butyl phthalate—can be explained by a combination of chemical physical properties and toxicologic potential. Given the phthalate exposures of children worldwide, the results from this study of Swedish children have global implications.

Airborne phthalate esters are present at detectable levels across the surface of Earth. They were first identified in outdoor urban air (Cautreels and Van Cauwenberghe 1976a, 1976b) and subsequently have been recognized as global pollutants (Atlas and Giam 1981; Giam et al. 1978) and major constituents of indoor air (Weschler 1980, 1984). Their presence in outdoor and indoor environments reflects their large emission rates coupled with moderate atmospheric lifetimes. The total global consumption of phthalate esters is estimated to exceed 3.5 million metric tons/year, with di(2-ethylhexyl) phthalate (DEHP) constituting roughly 50% of the market share (Cadogan and Howick 1996). Consumption of di-*n*-butyl phthalate (DnBP) and *n*-butyl benzyl (BBzP) phthalate is smaller but still quite large (> 100,000 metric tons/year each) (Cadogan and Howick 1996). Although DEHP plasticizes numerous products, roughly 95% of the current production is used in polyvinyl chloride (PVC) (National Toxicology Program 2003), where it typically constitutes 30% of PVC by weight (Cadogan and Howick 1996; Kavlock et al. 2002b). DnBP is used in latex adhesives, in nail polish and other cosmetic products, as a plasticizer in cellulose plastics, as a solvent for certain dyes, and, to a lesser extent than DEHP, as a plasticizer in PVC (Kavlock et al. 2002c). BBzP is a plasticizer for vinyl tile, carpet tiles, and artificial leather and is also used in certain adhesives (Kavlock et al. 2002a).

Research groups have assessed the exposures of various populations to phthalate esters by using their metabolites in human urine as biomarkers [Barr et al. 2003; Blount et al. 2000; Centers for Disease Control and Prevention (CDC) 2003; Koch et al. 2003]. The biomarker results translate to daily exposures for DnBP, BBzP, and DEHP of 1.5, 0.88, and 0.71 μg/kg/day in the United States (Kohn et al. 2000); 0.95, 0.71, and 0.84 μg/kg/day in the United States (derived from data from Barr et al. 2003, their Table 1, using the procedure outlined by Kohn et al. 2000); and 5.22, 0.60, and 13.8 μg/kg/day in Germany (Koch et al. 2003). These findings confirm the relatively large daily exposure to phthalates in industrialized countries. Although the dominant route of exposure to DnBP, BBzP, and DEHP is thought to be via ingestion (Fromme et al. 2004; Kavlock et al. 2002a, 2002b, 2002c), few if any population-based data are available to support this statement. Indeed, a recent study has demonstrated associations between phthalate concentrations in inhaled air and urinary monoester metabolites (Adibi et al. 2003).

The incidence of asthma and allergy has increased throughout the developed world over the past 30 years (Beasley et al. 2003). The short interval over which it has occurred implies that the increase is caused by changes in environmental exposures rather than genetic changes (Etzel 2003; Strachan 2000). Changes in indoor environments warrant special attention because indoor air constitutes a dominant exposure route. Increased exposures to allergens and/or adjuvants (enhancing factors) may each be partially responsible for the increase. Multidisciplinary reviews of the scientific literature on associations between indoor exposures and asthma and allergies (Ahlbom et al. 1998; Andersson et al. 1997; Bornehag et al. 2001; Schneider et al. 2003; Wargocki et al. 2002) indicate that the underlying causal factors responsible for these increases remain unknown.

The use of plasticized products and, consequently, exposures to phthalate esters have increased dramatically since the end of World War II. Phthalate esters have been suggested to act as either allergens or adjuvants (Jaakkola et al. 1999; Oie et al. 1997). Several recent studies have examined the ability of different phthalate esters to function as adjuvants in BALB/c mice injected with a known antigen. DEHP displayed an adjuvant effect with immunoglobulin G1 at a concentration of 2,000 mg/mL after both one and two boosters (Larsen et al. 2001b). In contrast, DnBP only showed an adjuvant effect with immunoglobulin G1 after the second booster (Larsen et al. 2002), and BBzP showed no adjuvant effect (Larsen et al. 2003). Consistent with these results, the monoester of DEHP showed an adjuvant effect whereas the monoesters of DnBP and BBzP did not (Larsen et al. 2001a).

The present study is a nested case–control study on 198 symptomatic children and 202 healthy controls, including detailed clinical examinations by physicians in parallel with extensive inspections and measurements within the subjects’ homes. The cases and controls were selected from the first phase (Dampness In Buildings and Health, phase I), which was a cross-sectional questionnaire soliciting health and environmental information regarding all 14,077 children 1–6 years of age in the county of Värmland, Sweden; responses were obtained for 10,852 (Bornehag et al. 2003).

The aim of the present study was to investigate potential associations between persistent allergic symptoms in children and the concentrations of different phthalates in dust collected from their homes.

## Materials and Methods

### Inclusion criteria for cases and controls.

The selection criteria for the cases (Dampness In Buildings and Health, phase II) were as follows: *a*) in the initial questionnaire, reports of at least two incidents of eczema, or wheezing or rhinitis without a cold, during the preceding 12 months; and *b*) in the follow-up questionnaire 1.5 years later, at least two of three possible symptoms reported. Inclusion criteria for the controls were *a*) no symptoms in the first questionnaire and *b*) no symptoms in the follow-up questionnaire. For both groups they had to *c*) not have rebuilt their homes because of moisture problems and *d*) not have changed residence since the first questionnaire. All children with at least two symptoms in the first questionnaire were invited to participate in the case–control study (*n* = 1,056, corresponding to 9.7% of the total population). In the first questionnaire, 5,303 (48.9%) reported no airway, eye, nose, or skin symptoms. Of these, 1,100 children were randomly selected and invited to participate in the case–control study. This process ultimately yielded 198 cases and 202 controls.

Families were more inclined to participate if the child was reported to have more symptoms, if there was no smoking in the family, and if they belonged to a higher socioeconomic group.

### Medical examination.

The medical examination of the 400 children (3–8 years of age) was performed during the same 2 weeks that the technical investigations of the homes, including dust collection, were carried out. Medical doctors examined the children and took a detailed history of each child. Blood samples were drawn from 387 children and screened for common allergens (Phadiatop, Pharmacia & Upjohn Diagnostics, Uppsala, Sweden), timothy, birch, mugworth, cat, horse, dog, house dust mites (*Dermatophagoides pteronyssinus* and *Dermatophagoides farinae*), and one mold (*Cladosporium*).

Physicians’ diagnoses of the children agreed well with the case–control status as reported in the questionnaire. All children with obvious asthma were found among cases, whereas 10 cases were found among controls (two children with rhinitis and eight children with eczema). Furthermore, 13 cases were found to be misclassified. In the analyses regarding case–control status, the study design has been used; that is, the 23 (10 plus 13) misclassified children have not been reclassified.

### Building investigations.

There were 10 pairs of siblings among the 400 children; hence, they lived in 390 buildings. Between October 2001 and April 2002, six professional inspectors performed visual inspections and indoor air quality assessments, including dust sampling, in these 390 dwellings. During these investigations, a preestablished checklist was followed regarding building characteristics, mold and water damages, surface materials, and other building-related items.

### Phthalates in dust.

Samples of dust from 390 homes were collected from molding and shelves in the children’s bedroom. The dust was collected on 90-mm membrane filters in holders made of styrene-acrylonitrile polymer mounted on a sampler made of polypropylene (VacuuMark disposable nozzle; Petersen Bach, Bjerringbro, Denmark) connected to a vacuum cleaner. The filter was weighed before and after sampling under controlled conditions. Conditioning the filters before weighing (23°C, 50% relative humidity) was critical to obtaining reproducible filter weights. From the 390 homes there were 9 missing samples, 13 samples with errors in the laboratory analysis, and 6 samples with a negative dust weight. Consequently, there were 362 valid samples. Only filters with a reliably measurable net increase in weight (≥ 25 mg) were included in the present analysis; 346 of the 362 dust samples met this criterion.

The dust samples were extracted in pre-cleaned 10-mL glass vials for 30 min using 2 mL dichloromethane. This procedure was repeated, and the two extracts were then combined and transferred to 3-mL autosampler vials. Aliquots from these vials were injected into either a gas chromatograph/mass selective detector (GC/MSD) for phthalate identification or a GC/flame ionization detector for quantitation. GC was performed using a 25-m capillary column (HP 1C; Agilent, Folsom, CA, USA; inner diameter, 0.2 mm; stationary phase, polydimethyl siloxane). The injector temperature was 280°C; column temperature started at 100°C for 3 min and then increased at 8°C/min to 300°C, which was maintained for 20 min. The detector temperature and transfer line to the MSD were maintained at 280°C. The analytical and field sampling techniques were tested in a preliminary study that found only limited influence from background contributions to the analyzed samples. In the present study, field blanks have indicated no significant background contributions. The dust concentrations (milligram per gram dust) of six phthalates were determined: diethyl phthalate (DEP), diisobutyl phthalate (DIBP), DnBP, BBzP, DEHP, and diisononyl phthalate (DINP).

### Statistical method.

The concentrations of phthalates in the dust were log-normally distributed. Hence, analyses of potential associations between concentrations of phthalates in dust and health outcomes have been conducted using nonparametric tests (Mann-Whitney *U*-test). Log-transformed, normally distributed concentrations were tested with parametric tests (*t*-test). The concentrations are reported as medians, as arithmetic means, and as geometric means with 95% confidence intervals (CIs). The CIs were calculated with a back-transform of mean log ± 2 × SE. Dose–response relationships were tested by factoring the phthalate concentrations into quartiles and using both uni- and multivariate logistic regression analyses. Adjustments have been made for environmental tobacco smoke as well as sex and age of the child, because these have been associated with asthma and allergic symptoms. Adjustments for type of building were made, because living in a privately owned single-family house was a selection factor for both cases and controls (Bornehag et al., unpublished data). Indeed, cases and controls lived mainly in single-family houses (88.7%). Furthermore, the frequency of PVC as flooring material was lower in single-family houses than in multifamily houses (51.6 vs. 71.8%). Adjustments for the construction period of the building and self-reported water leakage in the home during the previous 3 years were made because these are associated with the concentrations of phthalates in dust. Finally, adjustments were made for exposure to other phthalates. Multiple logistic regressions were performed by a backward elimination technique where only significant variables were included in the final model. The analyses were considered statistically significant when *p* < 0.05.

The study was approved by the local ethics committee.

## Results

Compared with other types of flooring materials, PVC flooring in the child’s bedroom was positively associated with case status [adjusted odds ratio (OR), 1.59; 95% CI, 1.05–2.41].

### Phthalates in dust.

**Figure 1:**
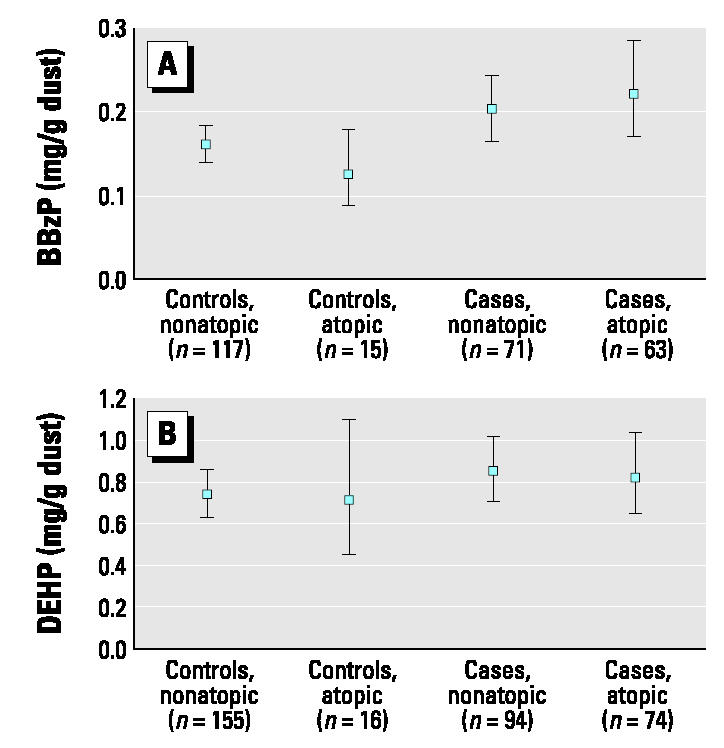
Geometric mean concentrations (95% CIs) of phthalates (*A*), BBzP, and (*B*), DEHP in surface dust from bedrooms of nonatopic and atopic children.

Results are presented in Tables 1–3 and Figure 1. In Tables 1 and 2, median phthalate dust concentrations are reported for data sets that include all valid samples with a reliably measurable net increase in weight (346 of 390 homes), and geometric mean concentrations are reported for data sets that exclude samples with phthalate dust concentrations less than the detection limit. (If, instead, nondetects were assigned concentrations of one-half the detection limit, then for phthalates with a large number of nondetects, their dust concentrations would no longer be log-normally distributed.) The geometric mean concentrations of BBzP and DEHP were higher in bedrooms with PVC flooring than in bedrooms without such flooring [BBzP: 0.208 (*n* = 164) vs. 0.147 (*n* = 107) mg/g dust; DEHP: 0.994 (*n* = 186) vs. 0.638 (*n* = 155) mg/g dust; both *p* < 0.001 by *t*-test]. DEP, DIBP, DnBP, and DINP were not associated with PVC flooring.

### Association between phthalates in dust and health effects.

Cases had a higher concentrations of BBzP in the dust samples from the children’s bedrooms than did the controls in parametric as well as in nonparametric tests (Table 1). Cases with physician-diagnosed rhinitis or eczema had higher BBzP concentrations in the bedroom dust compared with controls (Table 2). Furthermore, cases with doctor-diagnosed asthma had higher DEHP concentrations in the dust compared with controls. In analyses restricted to single-family and row houses, the same associations were found (data not shown).

In an analysis restricted to homes with PVC flooring in the child’s bedroom (*n* = 189), the median BBzP concentration was significantly higher among cases compared with controls (0.21 vs. 0.16 mg/g dust, respectively; Mann-Whitney *U*-test, *p* = 0.042), and BBzP was associated with rhinitis and eczema (Table 2). Such differences between cases and controls were not observed for DEHP.

BBzP concentrations in the highest quartile were associated with an increased risk of being a “case child” (Table 3). The same association was found after adjusting for possible confounders. Table 3 also shows associations between phthalates in dust and doctor-diagnosed asthma, rhinitis, or eczema. A dose–response relationship was found between concentrations of BBzP in dust and doctor-diagnosed rhinitis and eczema in both crude and adjusted analyses. For DEHP, a dose–response relationship was found for asthma in both crude and adjusted analyses, as well as in analysis restricted to single-family houses (data not shown for the latter).

### Specific immunoglobulin E in blood.

Figure 1 presents the concentration of phthalates in dust among cases and controls with and without specific immunoglobulin E in blood (i.e., atopics and nonatopics). Within the group of cases, the highest geometric mean concentrations of BBzP were found in dust from the bedrooms of atopics. However, when comparing cases with and without atopy, the difference was not statistically significant (*p* = 0.564).

## Discussion

In the present study we found associations between dust concentrations of specific phthalate esters and asthma, rhinitis, and eczema. As shown in Tables 2 and 3, BBzP is significantly associated with doctor-diagnosed rhinitis and eczema, whereas DEHP is significantly associated with doctor-diagnosed asthma. Interestingly, no such associations are found for DnBP despite the fact that the median concentrations of BBzP and DnBP in the settled dust were comparable (0.150 vs. 0.135 mg/g; Table 1). Hence, these three phthalates display strikingly different associations between their dust concentrations and the health outcomes monitored in this study. From a physical chemistry viewpoint, DnBP, BBzP, and DEHP are significantly different from one another; they possess different vapor pressures, polarities, water solubilities, and octanol/air partition coefficients. For example, the vapor pressures of DnBP and BBzP are two orders of magnitude greater than that of DEHP. This means that greater fractions of DnBP and BBzP are in the gas phase as opposed to the condensed phase (i.e., associated with dust and airborne particles). We estimate that, for a particle concentration of 20 μg/m^3^, > 80% of airborne DnBP and > 80% of airborne BBzP are in the gas phase, whereas > 85% of airborne DEHP is associated with airborne particles (Weschler 2003). The deposition of a compound in the respiratory tract is strongly influenced by whether it is present in the gas phase or associated with airborne particles. Furthermore, as a consequence of their inherent chemical differences, DnBP, BBzP, and DEHP, as well as their monoester metabolites, produce different effects in a mouse model (Larsen et al. 2001a, 2001b, 2002, 2003). Furthermore, each of these phthalates has its distinct human metabolic pathway (Barr et al. 2003). We suspect that the different relative distributions between the gas and condensed phases, coupled with different toxicologic and pharmacokinetic behaviors, contribute to the fact that DnBP, BBzP, and DEHP are associated with different health outcomes (i.e., DnBP, no associations; BBzP, skin and mucosa symptoms; DEHP, lower airway symptoms).

In the present study there is a general association between PVC flooring and case status (OR, 1.59). Both BBzP and DEHP correlate with the amount of PVC flooring in the subjects’ homes. However, these two phthalates are not associated with health effects simply because they are associated with PVC flooring. This conclusion is supported by a number of observations: First, specific associations between BBzP and DEHP dust concentrations and doctor-diagnosed diseases (Table 3) are more pronounced than associations between PVC flooring and such diseases. Second, although BBzP and DEHP dust concentrations do correlate, the correlation is weak (*R* = 0.52), and they are associated with different health effects. Third, in a restricted analysis, including only homes with PVC flooring, higher concentrations of BBzP were found in dust from case homes than in that from control homes.

The reported concentrations of phthalates in the bedroom dust (Table 1) are consistent with those reported in other studies. In dust samples from 120 U.S. homes located on Cape Cod, Massachusetts (Rudel et al. 2003), the median concentrations were 0.34, 0.045, and 0.020 mg/g dust for DEHP, BBzP, and DnBP, respectively. In a study of 59 apartments in Berlin, Germany (Fromme et al. 2004), the median concentrations were 0.70, 0.030, and 0.047 mg/g dust for DEHP, BBzP, and DnBP. Clausen et al. (2003) measured mean DEHP concentrations of 3.2 mg/g dust in 15 Danish schools and 0.86 mg/g dust for 23 Danish homes. Oie et al. (1997) reported mean concentrations of 0.64 mg DEHP/g dust and 0.11 mg BBzP/g dust for 38 homes in Norway. Pohner et al. (1997) reported a 95th percentile DEHP concentration of 2.0 mg/g dust for 272 German homes, whereas another German study on 286 homes reported a 95th percentile DEHP concentration of 2.6 mg/g dust (Butte et al. 2001).

Regarding atopic status and its association with phthalate dust concentrations, the chosen study design is not optimal. Because there were only 16 atopic controls, the power of the analysis on atopic children is limited. On the other hand, our findings could be interpreted to mean that the mechanism is of a non-immunologic nature (e.g., exposure increases the risk for irritation).

To identify potential selection biases in the study group, we obtained information for all invited families from the first cross-sectional questionnaire. This revealed that the final study group contained significantly more single-family houses than the eligible population. Adjusting and restricting the analyses have addressed this problem. There was no selection bias regarding PVC flooring because included and nonincluded cases and controls reported about the same frequency of occurrence of PVC flooring in the child’s bedroom (Bornehag et al., unpublished data). Furthermore, 10 controls and 13 cases were misclassified when comparing self-reported symptoms and doctors diagnoses. However, when these children were excluded from the analyses, the reported associations remained. Finally, to be included as a “case,” a child was required to have at least two symptoms. Consequently, this study was not fine-tuned to examine associations between building factors and single symptoms (i.e., asthma, rhinitis, or eczema). However, even if the design is suboptimal, meaning it was more difficult to find associations between single symptoms and exposures, the association between selected building factors and single symptoms is meaningful and possibly underestimates true associations.

The reported analyses are based on samples with a weight > 25 mg. However, when including all samples (*n* = 362), the reported associations between exposure and symptoms remained or became stronger (data not shown).

Koo et al. (2002) present weak associations between exposure estimates for different phthalate esters, based on their urinary biomarkers, and the level of education, family income, and residency (urban or rural) in a reference U.S. population. Given that study, one might speculate that the associations reported in the present study are driven by demographic factors. However, in contrast to the United States, where 22.4% of the children live in households with incomes < 50% of the national median, in Sweden only 2.6% of the children live in such households (Unicef 2000). Additionally, the association in our study holds when the analysis is restricted to single-family houses; such homes have an even more homogeneous socioeconomic status. Hence, different demographic factors between cases and controls appear to be an unlikely explanation for the associations observed in the present study. Furthermore, given that the dust concentrations of DnBP, BBzP, and DEHP display quite different associations with different symptoms, the associations reflect a biologic response rather than just lifestyle or demographic factors associated with an increased use of plasticized materials.

This study demonstrates associations between BBzP and DEHP concentrations in dust and selected allergies and asthma. Although multiple factors likely are responsible for the increases in allergies and asthma that have been documented in developed countries over the past 30 years, it is striking that these increases have occurred during a period when plasticized products have become ubiquitous in the homes, schools, and workplaces of the developed world.

## Figures and Tables

**Table 1:** Concentrations of phthalates in surface dust from children’s bedrooms.

		Median (arithmetic mean) concentration of phthalates (mg/g dust)						Cases		Controls		
Phthalate	No. of homes*^a^*	All samples (*n* = 346)	Cases (*n* = 175)*^b^*	Controls (*n* = 177)*^b^*	*U*-test*^c^* (*p*-value)	No. of homes*^d^*	All samples GM conc	No.	GM conc [(95% CI) mg/g dust]	No.	GM conc [(95% CI) mg/g dust]	*t*-Test*^e^* (*p*-value)
DEP	346	0.000 (0.031)	0.000 (0.046)	0.000 (0.018)	0.628	47	0.073	22	0.102 (0.049–0.211)	26	0.058 (0.035–0.097)	0.200
DIBP	346	0.045 (0.097)	0.042 (0.102)	0.048 (0.092)	0.424	290	0.056	141	0.058 (0.048–0.070)	154	0.055 (0.046–0.065)	0.635
DnBP	346	0.150 (0.226)	0.150 (0.228)	0.149 (0.220)	0.914	308	0.174	158	0.171 (0.152–0.193)	154	0.178 (0.157–0.201)	0.639
BBzP	346	0.135 (0.319)	0.152 (0.472)	0.118 (0.163)	0.014	272	0.181	139	0.209 (0.180–0.244)	137	0.157 (0.139–0.178)	0.004
DEHP	346	0.770 (1.310)	0.828 (1.384)	0.723 (1.229)	0.160	343	0.789	173	0.836 (0.724–0.964)	176	0.741 (0.643–0.855)	0.232
DINP	346	0.041 (0.639)	0.000 (0.671)	0.047 (0.589)	0.848	175	0.451	87	0.453 (0.352–0.583)	90	0.446 (0.351–0.566)	0.925

Abbreviations: conc, concentration; GM, geometric mean.

a Number of homes with a dust sample weight > 25 mg.

b The sum of cases and controls is 352 because, among the 346 bedrooms, there were six bedrooms shared by siblings.

c Mann-Whitney *U*-test.

d Number of homes with a dust sample weight > 25 mg and a phthalate concentration greater than the detection limit (0.040 mg/g dust for DnBP, BBzP, and DEHP).

e Test of the difference between cases and controls made on mean log-transformed concentration.

**Table 2:** Concentration of phthalates (BBzP and DEHP) in surface dust for case children with a doctor-diagnosed disease compared with controls.

		Cases*^a^*		Controls			Cases		Controls		
Phthalate	Disease	No.	Median conc (mg/g dust)	No.	Median conc (mg/g dust)	*U*-test*^b^* (*p*-value)	No.	GM conc [(95% CI) mg/g dust]	No.	GM conc [(95% CI) mg/g dust]	*t*-Test*^c^* (*p*-value)
All homes											
BBzP	Asthma	106	0.152	177	0.118	0.064	82	0.219 (0.177–0.270)	137	0.157 (0.139–0.178)	0.005
	Rhinitis	79	0.181	177	0.118	0.007	65	0.237 (0.185–0.304)	137	0.157 (0.139–0.178)	0.001
	Eczema	115	0.181	177	0.118	0.001	95	0.224 (0.186–0.269)	137	0.157 (0.139–0.178)	0.001
DEHP	Asthma	106	0.899	177	0.723	0.008	106	0.966 (0.807–1.156)	176	0.741 (0.643–0.855)	0.022
	Rhinitis	79	0.783	177	0.723	0.383	78	0.811 (0.638–1.030)	176	0.741 (0.643–0.855)	0.510
	Eczema	115	0.844	177	0.723	0.111	115	0.855 (0.721–1.014)	176	0.741 (0.643–0.855)	0.207
Homes with PVC flooring in the child’s bedroom											
BBzP	Asthma	59	0.195	82	0.159	0.168	52	0.237 (0.177–0.316)	71	0.177 (0.148–0.212)	0.076
	Rhinitis	45	0.216	82	0.159	0.008	43	0.265 (0.192–0.366)	71	0.177 (0.148–0.212)	0.018
	Eczema	70	0.216	82	0.159	0.003	66	0.257 (0.204–0.324)	71	0.177 (0.148–0.212)	0.011
DEHP	Asthma	59	1.006	82	0.855	0.149	59	1.148 (0.904–1.459)	82	0.938 (0.752–1.169)	0.228
	Rhinitis	45	0.792	82	0.855	0.924	44	1.040 (0.771–1.403)	82	0.938 (0.752–1.169)	0.586
	Eczema	70	0.904	82	0.855	0.379	70	1.045 (0.845–1.291)	82	0.938 (0.752–1.169)	0.491

Abbreviations: conc, concentration; GM, geometric mean.

a Cases with doctor diagnosed disease (asthma, rhinitis, or eczema).

b Mann-Whitney *U*-test.

c Test of the difference between cases and controls made on mean log-transformed concentration.

**Table 3:** Crude and adjusted ORs (95% CIs) between phthalates (BBzP and DEHP) in surface dust and case status or doctor-diagnosed disease.

	Quartile				
Group*^a^*	1 (ref; *n* = 88)	2 (*n* = 88)	3 (*n* = 88)	4 (*n* = 88)	*p*-Value*^b^*
BBzP					
Ranges (mg BBzP/g dust)	0.00–0.05	0.05–0.13	0.13–0.25	0.25–45.55	
Crude analysis					
Case status	1.0	0.69 (0.38–1.26)	1.00 (0.55–1.81)	2.01 (1.10–3.69)	0.012
Asthma	1.0	0.63 (0.31–1.27)	0.59 (0.45–1.76)	1.92 (0.98–3.79)	0.039
Rhinitis	1.0	0.85 (0.38–1.89)	1.12 (0.51–2.47)	2.69 (1.26–5.76)	0.006
Eczema	1.0	0.74 (0.36–1.52)	1.44 (0.73–2.81)	2.52 (1.26–5.00)	0.002
Adjusted*^c^* analysis					
Case status	1.0	0.77 (0.40–1.46)	1.01 (0.53–1.90)	1.95 (1.02–3.74)	—
Asthma	1.0	0.67 (0.33–1.38)	0.88 (0.43–1.80)	1.87 (0.92–3.81)	—
Rhinitis	1.0	1.03 (0.44–2.39)	1.23 (0.53–2.88)	3.04 (1.34–6.89)	—
Eczema	1.0	0.84 (0.40–1.76)	1.45 (0.71–2.97)	2.56 (1.24–5.32)	—
DEHP					
Ranges (mg DEHP/g dust)	0.00–0.46	0.46–0.77	0.77–1.30	1.30–40.46	
Crude analysis					
Case status	1.0	0.91 (0.50–1.65)	1.05 (0.58–1.89)	1.44 (0.80–2.61)	0.199
Asthma	1.0	1.11 (0.53–2.31)	1.51 (0.74–3.07)	2.36 (1.17–4.75)	0.009
Rhinitis	1.0	1.12 (0.53–2.36)	0.96 (0.44–2.11)	1.55 (0.73–3.28)	0.331
Eczema	1.0	1.00 (0.50–1.97)	1.35 (0.70–2.62)	1.50 (0.76–2.96)	0.161
Adjusted*^c^* analysis					
Case status	1.0	NS	NS	NS	—
Asthma	1.0	1.56 (0.70–3.46)	2.05 (0.94–4.47)	2.93 (1.36–6.34)	—
Rhinitis	1.0	NS	NS	NS	—
Eczema	1.0	NS	NS	NS	—

— , no analyses have been done because linear-by-linear association cannot be done in a multivariate manner; NS, not significant in model, using backward elimination; ref, reference.

a Case status and subgroups with asthma, rhinitis, or eczema compared with controls.

b Linear-by-linear association.

c Adjustments made for sex, age, smoking at home, type of building, construction period, self-reported flooding during preceding 3 years, and the other phthalate variable (in quartiles), using backward elimination method; only significant variables were included in the final model.
